# Treadmill running exercise prevents senile osteoporosis and upregulates the Wnt signaling pathway in SAMP6 mice

**DOI:** 10.18632/oncotarget.12125

**Published:** 2016-09-19

**Authors:** Xi Chen, Lihui Li, Jianmin Guo, Lingli Zhang, Yu Yuan, Binglin Chen, Zhongguang Sun, Jiake Xu, Jun Zou

**Affiliations:** ^1^ School of Kinesiology, Shanghai University of Sport, Shanghai, P. R. China; ^2^ School of Sports Science, Wenzhou Medical University, Wenzhou, P. R. China; ^3^ School of Pathology and Laboratory Medicine, The University of Western Australia, Western Australia, Australia

**Keywords:** exercise, osteoporosis, Wnt signaling pathway, SAMP6, senile, Gerotarget

## Abstract

This study examined the effects of different exercise intensities and durations on bone mineral density (BMD) and bone strength in senescence-accelerated mouse prone 6 (SAMP6) and determined the involvement of the Wnt signaling pathway in exercise-induced osteogenesis. Three-month-old male SAMP6 mice were randomly assigned to different speeds of treadmill running exercise representing low, medium and high intensity, with the duration of five and nine weeks, respectively. We showed that medium-intensity exercise had positive effects on skeletal health, including BMD and bone strength, and the efficacy was higher than that of low-intensity exercise. Interestingly, high-intensity exercise can maintain or even increase bone strength, despite its negative effects on bone mass. Nine weeks of exercise was superior to 5 weeks of exercise, particularly for low-intensity exercise. Furthermore, these effects of exercise-induced osteogenesis are accompanied by activation of the Wnt signaling pathway. Taken together, these results suggest that the positive effects of exercise on osteoporosis prevention are intensity and duration-dependent, and may involve the regulation of Wnt signaling pathways.

## INTRODUCTION

The balance between bone formation and resorption maintains adult bone and skeletal health [1]. Disruption of the balance in bone homeostasis leads to the development of bone-related diseases, particularly osteoporosis, which decreases bone mineral density (BMD), deteriorates bone micro-architecture, and increases the risk of fracture [2–4]. Osteoporosis has become the most common disease in aging population, particularly in menopausal women [5, 6]. The treatment and prevention of osteoporosis have been widely studied, but there is no perfect anti-osteoporosis drug [7–9]. Appropriate exercise is one of the most important positive stimuli for bone formation, and it appears to have positive effects on the prevention of osteoporosis [10, 11]. Exercise improves bone mass, bone geometry, and, consequently, bone strength in older individuals and may also improve bone quality without affecting bone mass. Thus, exercise has been recommended as an appropriate life style intervention or physical therapy for osteoporosis prevention [12, 13]. However, other studies have demonstrated that exhaustive or strenuous exercise increases bone resorption and leads to stress-related fractures among military recruits and elite athletes [14]. Thus, the effect of exercise on bone health appears to be dependent on the intensity and duration. However, the extent to which intensity and duration affect bone mass and strength remains unclear.

Exercise increases serum levels of sex hormones in mice and decreases levels of cytokines, e.g., IL-1, IL-6 and Cox-2, which inhibit osteoclastogenesis and bone resorption [15, 16]. Mechanical loading also contributes to exercise-induced increases in bone mass and strength [17], and signaling pathways such as BMPs, MAPK and Wnt may be involved in osteogenesis [18]. The Wnt/β-catenin signaling pathway is an important pathway in bone anabolic activity that positively modulates bone mass by regulating mesenchymal precursor commitment to the osteoblast lineage [18–20], enhancing osteoblast proliferation and terminal differentiation [21–23]. However, few studies have examined the effects of exercise intensity and duration on activation of the Wnt signaling pathway, and their relationship with the prevention of senile osteoporosis.

The senescence-accelerated mouse strain (SAMP) has a short life span and strain-specific pathological features and has been accepted as an experimental animal model for age-associated disease research in systematically designed studies [24]. SAMP6 has been used as a murine model of senile osteoporosis because it has low bone mass in the vertebra, tibia and femur [25, 26]. High levels of secreted frizzled-related protein (Sfrp4) in SAMP6 mice negatively regulate bone formation via inhibition of the Wnt signaling pathway [27], and inadequate osteoblast renewal in SAMP6 mice can be reversed by lithium chloride feeding via upregulating Wnt signaling pathway [28].

Therefore, the present study used SAMP6 mice as a model of senile osteoporosis to examine the effects of different intensities and durations of running exercise on bone mass and strength and evaluated whether the Wnt signaling pathway is involved in exercise-induced bone remodeling.

## RESULTS

### Animal weights

**Figure 1:**
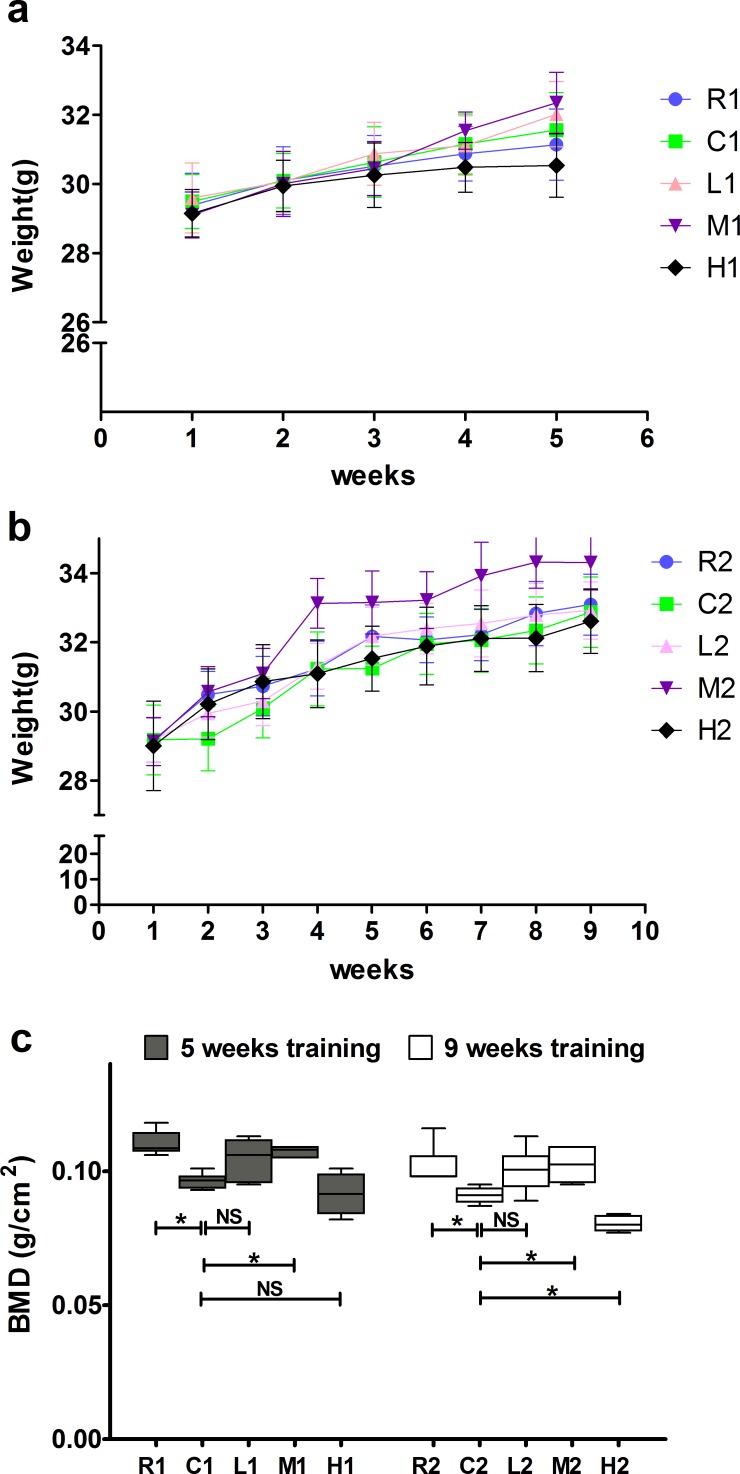
Effects of exercise on body weight and bone mineral density **a.**, **b.** Body weight of mice in each group. **c.** Bone mineral density of mice in each group. Data are represented as mean +/− SD (*n* = 6). *: *P* < 0.05. R, SAMR1 non-exercise group; C, SAMP6, non-exercise group; L, SAMP6, low-intensity group; M, SAMP6, medium-intensity group; H1, SAMP6, high-intensity group.

All mice exhibited a slight increase in weight during the experimental period, but there were no significant differences among different groups in the same week (Figure 1a, 1b).

### Effect of exercise on BMD

As shown in Figure 1c, the BMDs were significantly higher in the M and R groups than in the C groups after both 5 and 9 weeks of exercise training, whereas no significant differences were detected between the C and L groups, suggesting that exercise-induced increased BMD was mainly dependent on intensity but not duration. In addition, the BMD was significantly lower in the H2 group than in the C2 group after 9 weeks of exercise training, but no significant differenc wasobserved between the H1 and C1 groups after 5 weeks oftraining, indicating that long term but not short term of high-intensity exercise decreases BMD.

### Effects of exercise on bone biomechanics

**Figure 2:**
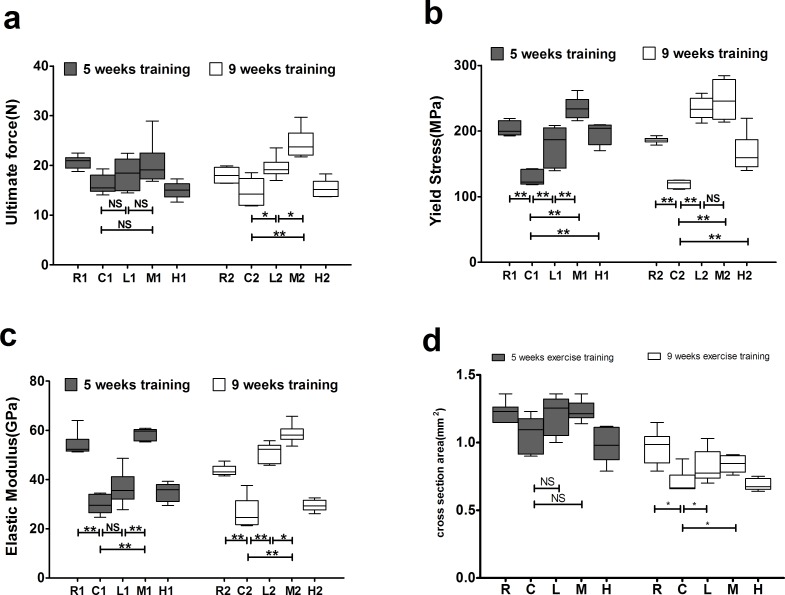
Effects of exercise on bone biomechanics **a.** Effects of exercise on bone ultimate force. **b.** Effects of exercise on bone yield stress. **c.** Effects of exercise on bone elastic modulus. **d.** Effects of exercise on cross sectional area. Data are represented as mean +/− SD (*n* = 6). *: *P* < 0.05, **: *P* < 0.01. R, SAMR1 non-exercise group; C, SAMP6, non-exercise group; L, SAMP6, low-intensity group; M, SAMP6, medium-intensity group; H1, SAMP6, high-intensity group.

The M2 and L2 groups exhibited significantly higher ultimate force values than the C2 group after 9 weeks of exercise training, and the M2 group had a significantly higher valuethan the L2 group, whereas no significant differences were observed among the groups after 5 weeks of training (Figure 2a).

The R, L, M and H groups had significantly higher yield stress than the C groups after both 5 and 9 weeks of exercise training. The M1 group had significantly higher yield stress than the L1 group after 5 weeks of training, whereas no significant differences were detected between the M2 and L2 groups after 9 weeks of training (Figure 2b).

The R and M groups had significantly greater elastic moduli than the C groups after both 5 and 9 weeks of running exercise, and the M groups had significantly greater elastic moduli than the L groups (Figure 2c). In addition, the L2 group had a significantly higher elastic modulus than the C2 group after 9 weeks of training, whereas no significant difference was detected between the L1 and C1 groups after 5 weeks of training (Figure 2c).

The R groups had significantly greater cross sectional area (CSA) than the C groups after both 5 and 9 weeks of exercise training. The M2 and L2 groups had significantly higher CSA than C2 group after 9 weeks of training, with no significantly differences observed between M1 and C1 groups after 5 weeks of training (Figure 2d).

These results suggest that exercise improves bone strength depending on intensity and duration, and the increased CSAs in response to exercise training promote the bone strength. Medium intensity of exercise is more effective on increased bone strength than low intensity of exercise, and 9 weeks of exercise is better than that of 5 weeks. Yet combined with medium intensity and 9-week exercise has no greater efficacy in bone strength when compared to 5-week medium exercise, while long duration of low intensity exercise has greater efficacy than short duration.

### Effects of exercise on bone histomorphometric parameters

As shown in Table 1, the trabecular bone volume (BV/TV), mineralizing surface (MS/BS), osteoid thickness (O.Th) and bone formation rate (BFR) were significantly elevated in the R1 and R2 groups compared with the C1 and C2 groups, respectively. The mineral apposition rate (MAR) was significantly higher in the R1 group than in the C1 group, and the bone cortex mineralization rate (mAR) was significantly higher in R2 group than in the C2 group.

**Table 1:** Effect of exercise on bone histomorphometric parameters

Parameter	5 weeks of exercise					9 weeks of exercise				
	R1	C1	L1	M1	H1	R2	C2	L2	M2	H2
MAR(μm/d)	1.73±0.11	1.4±0.88**	1.49±0.14	1.59±0.11	1.41±0.16	1.56±0.16	1.39±0.08	1.60±0.09^▲^	1.69±0.09^▲▲^	1.28±0.12
BV/TV(%)	9.37±0.83	7.23±1.21**	8.51±0.73	9.30±0.76^∆∆^	5.84±1.07	8.36±0.53	6.67±0.55	7.85±1.52	9.10±1.09^▲^	5.57±1.55
MS/BS(%)	5.99±0.56	3.95±0.25**	4.26±1.12	5.23±0.88	3.56±0.73	5.43±0.54	3.42±0.64^##^	4.49±0.74^▲^	6.10±0.72^▲▲ ■■^	2.79±0.23
ES/BS(%)	5.58±0.92	5.66±1.04	5.04±0.75	5.59±1.33	5.66±0.89	4.83±0.63	4.74±0.76	4.03±0.77	5.53±1.01^■^	4.64±0.4
mAR(μm/d)	1.83±0.17	1.45±0.16	1.65±0.28	1.81±0.18	1.43±0.37	1.71±0.14	1.33±0.19^#^	1.71±0.14^▲^	1.92±0.24^▲ ▲^	1.33±0.34
oTh(μm)	8.54±0.99	6.45±1.26*	6.82±1.42	7.43±0.79	6.63±1.05	8.28±0.68	6.09±0.74^##^	6.54±0.86	6.81±1.06	6.19±1.11
BFR(μm/d)	5.9±0.84	3.73±0.82**	3.82±1.03	6.04±0.81^∆∆ □□^	3.54±0.62	5.44±0.83	3.11±1.33^##^	4.40±1.28	6.60±0.89^▲▲^	1.98±0.57

Abbreviations: BV/TV, bone volume; MAR, mineral apposition rate; MS/BS, mineralizing surface; ES/BS, eroded surface; mAR, bone cortex mineralization rate; O.Th, osteoid thickness; BFR, bone formation rate. Compared with R1:*P < 0.05, **P < 0.01, Compared with R2: ^#^p < 0.05, ^##^*P* < 0.01; Compared with C1:^∆^*P* < 0.05, ^∆∆^*P* < 0.01; Compared with C2:^▲^*P* < 0.05, ^▲▲^*P* < 0.01; Compared with L1: ^□^*P* < 0.05, ^□□^*P* < 0.01; Compared with L2:^■^*P* < 0.05, ^■■^*P* < 0.01;R, SAMR1 non-exercise group; C, SAMP6, non-exercise group; L, SAMP6, low-intensity group; M, SAMP6, medium-intensity group; H1, SAMP6, high-intensity group.

The BV/TV, MAR, MS/BS, mAR and BFR were significantly higher in the M2 group than in the C2 group, whereas only the BV/TV and BFR were significantly increased in the M1 group compared with the C1 group, and no other significant differences were observed between the M1 and C1 groups, suggesting that 5 weeks of medium-intensity exercise increases trabecular bone formation and 9 weeks of such exercise increases both trabecular and cortical bone formation. Additionally, the L2 group exhibited significantly increases in the MAR, MS/BS, and mAR compared with the C2 group after 9 weeks of exercise training, but no significant differences were detected between the L1 and C1 groups after 5 weeks of training, indicating that only a long term of low intensity exercise increased bone formation. Furthermore, no significant difference in the eroded surface (ES/BS) was detected between the L and M groups and the C groups after both 5 and 9 weeks of training.

### Changes in Wnt1, LRP5 and β-catenin mRNA expression in the right tibia

**Figure 3:**
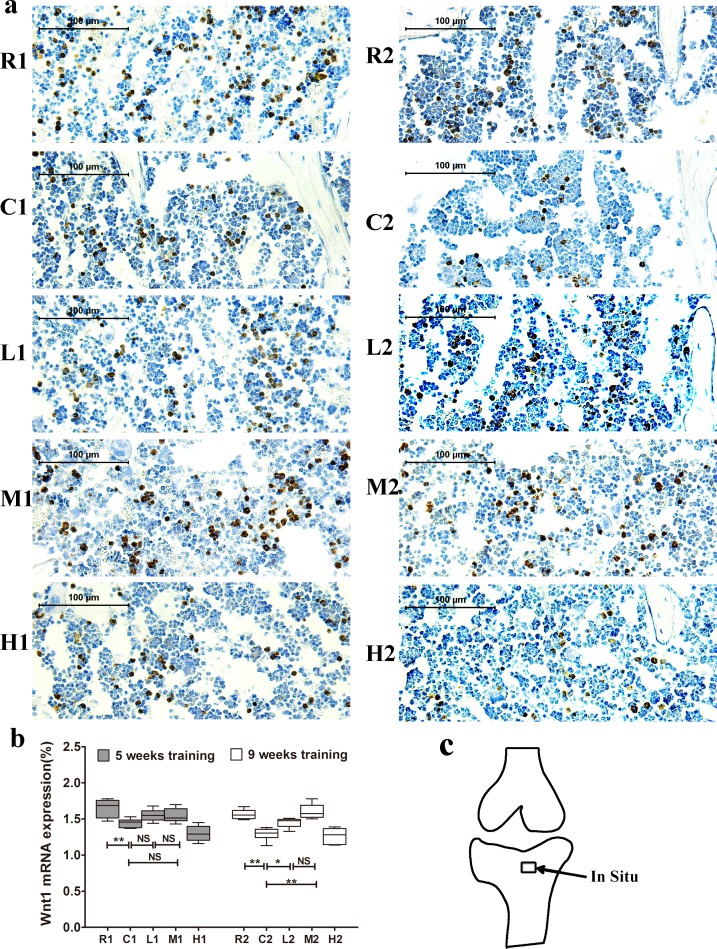
Changes of Wnt1 mRNA expression in the left tibia **a.** Representative images of Wnt1 mRNA expression by in situ hybridization in each group. The positive cells stained by brown color. **b.** Wnt1 mRNA expression. Percentage of positive cells were calculated from more than five high-power fields (× 400). Data are represented as mean +/− SD (*n* = 6). *: *P* < 0.05, **: *P* < 0.01. **c.** Location of in situ hybridization. R, SAMR1 non-exercise group; C, SAMP6, non-exercise group; L, SAMP6, low-intensity group; M, SAMP6, medium-intensity group; H1, SAMP6, high-intensity group.

We next examined Wnt1, LRP5 and β-catenin mRNA expression in the tibia via *in situ* hybridization staining. Wnt1 expression was significantly lower in the C1 and C2 groups than in the R1 and R2 groups, respectively, and it was significantly higher in the M2 and L2 groups than in the C2 group after 9 weeks of exercise training. Further, no significant differences were observed between the M1 and L1 groups and the C1 group after 5 weeks of training (Figure 3).

**Figure 4:**
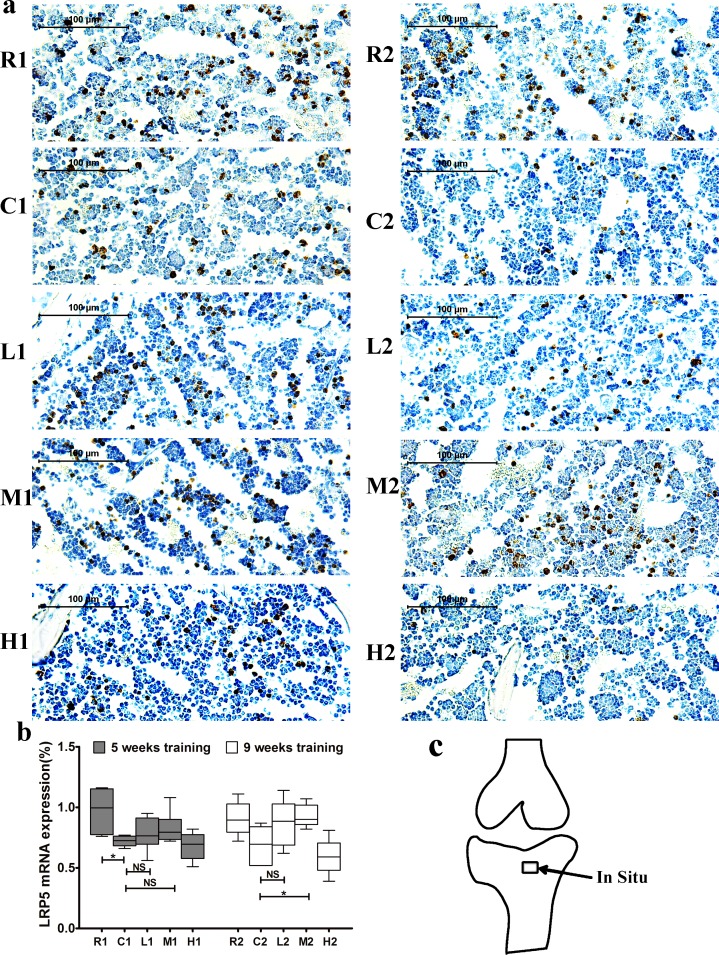
Changes of Lrp5 mRNA expression in the left tibia **a.** Representative images of Lrp5 mRNA expression by in situ hybridization in each group. The positive cells stained by brown color. **b.** Lrp5 mRNA expression. Percentage of positive cells were calculated from more than five high-power fields (× 400). Data are represented as mean +/− SD (*n* = 6). *: *P* < 0.05, **: *P* < 0.01. **c.** Location of in situ hybridization. R, SAMR1 non-exercise group; C, SAMP6, non-exercise group; L, SAMP6, low-intensity group; M, SAMP6 , medium-intensity group; H1, SAMP6, high-intensity group.

Lrp5 expression was significantly lower in the C1 group than in the R1 group, and it did not differ between the C2 and R2 groups. In addition, its expression was significantly increased in the M2 group compare with the C2 group after 9 weeks of exercise training, and no significant difference was observed between the M1 and C1 groups (Figure 4).

**Figure 5:**
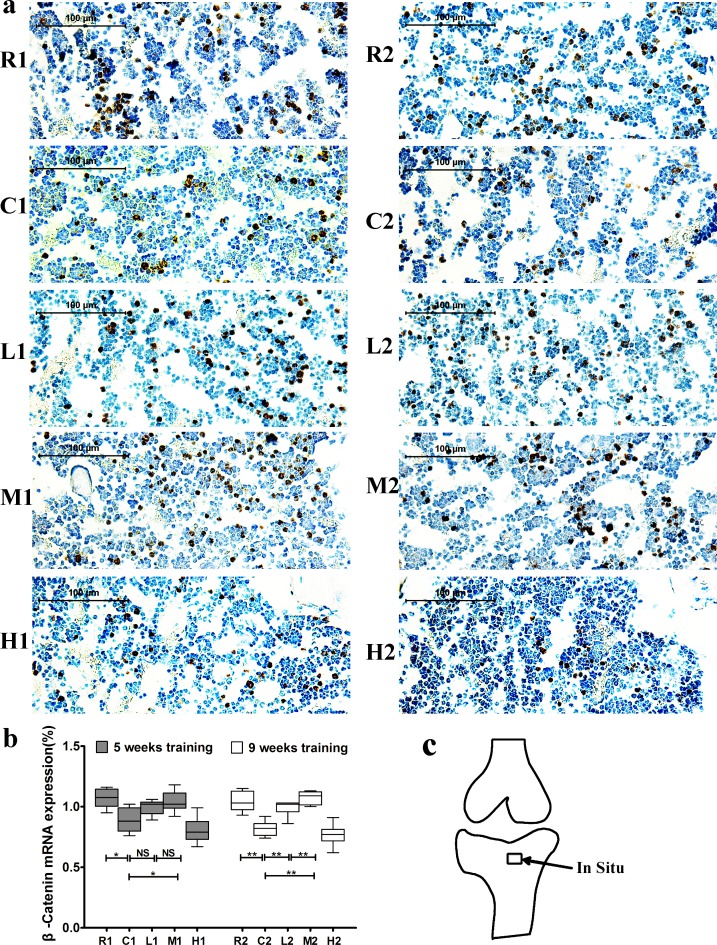
Changes of β-catenin mRNA expression in the left tibia **a.** Representative images of β-catenin mRNA expression by in situ hybridization in each group. The positive cells stained by brown color. **b.** β-catenin mRNA expression. Percentage of positive cells were calculated from more than five high-power fields (× 400). Data are represented as mean +/− SD (*n* = 6). *: *P* < 0.05, **: *P* < 0.01. **c.** Location of in situ hybridization. R, SAMR1 non-exercise group; C, SAMP6, non-exercise group; L, SAMP6, low-intensity group; M, SAMP6, medium-intensity group; H1, SAMP6, high-intensity group.

β-catenin mRNA expression was significantly lower in the C groups than in the R groups but was significantly higher in the M groups than in the C groups after both 5 and 9 weeks of exercise training. Additionally, its expression was significantly higher in the L2 group than in the C2 group, and no significant difference was observed between the L1 and C1 groups (Figure 5). Sense probes as negative controls did not detect any signal (data not shown). Collectively, our data suggest that exercise-induced upregulation of Wnt1 mRNA expression is dependent on exercise duration but not intensity, while Lrp5 and β-catenin expression are dependent on both the duration and intensity.

### Effects of exercise on serum levels of ALP, OCN and PTH

**Figure 6:**
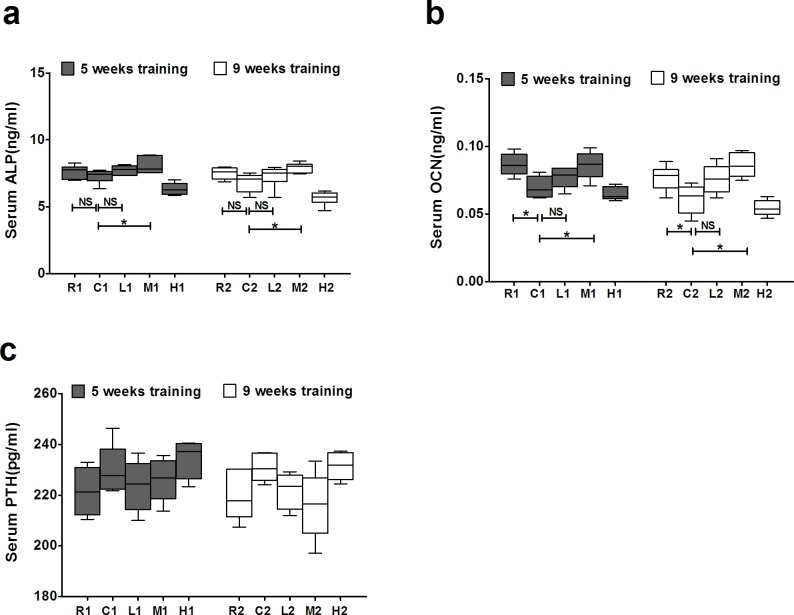
Effects of exercise on serum levels of ALP, OCN and PTH in each group **a.** The serum levels of ALP. **b.** The serum levels of OCN. **c.** The serum levels of PTH. Data are represented as mean +/− SD (*n* = 6). *: *P* < 0.05, **: *P* < 0.01. R, SAMR1 non-exercise group; C, SAMP6, non-exercise group; L, SAMP6, low-intensity group; M, SAMP6, medium-intensity group; H1, SAMP6, high-intensity group.

We then analyzed the serum levels of alkaline phosphatase (ALP) and osteocalcin (OCN), which reflect the level of bone modeling, particularly that of osteoblastic bone formation. The serum OCN levels were significantly lower in the C groups than in the R groups, whereas the M groups had significantly higher serum levels of ALP and OCN than the C groups in both the 5-week and 9-week exercise training groups. However, no significant difference in the ALP or OCN level was observed between the L and C groups (Figure 6a, 6b). These results indicate that exercise increases serum level of ALP and OCN in an intensity but not duration dependent manner, leading to the anabolic effect of bone for the prevention of senile osteoporosis.

The level of parathyroid hormone (PTH), a positive regulator of bone resorption, did not differ among all of the groups (Figure 6c), indicating that the beneficial effect of exercise on preventing osteoporosis was not attributed to a change in the serum PTH level.

## DISCUSSION

The present study demonstrated that SAMP6 mice had lower BMD and bone strength than strain SAM/resistant 1 (SAMR1) mice with aging after 3 months, leading to a reduction in resistance to bending fractures. In addition, low bone formation was observed in SAMP6 mice, indicating that SAMP6 mice are a good model of senile osteoporosis.

The results of this study showed that running exercise increased BMD and bone strength in SAMP6 mice, and these changes were beneficial for the prevention of senile osteoporosis. Our findings are consistent with Ishihara's report [29], showing that treadmill running exercise prevents age-associated osteoporosis in SAMP6 mice, and are further supported by bone biomechanical and histomorphometric parameters and serum levels of ALP and OCN. Furthermore, medium-intensity exercise increased the BV/TV, MS/BS, BFR and serum ALP and OCN levels, but these values did not significantly change after low-intensity exercise, indicating that exercise intensity plays an essential role in the positive effects on bone formation. However, high-intensity exercise decreased the BMD in the SAMP6 mice, particularly after 9 weeks of training. This result is likely due to the inhibition of bone formation without affecting bone resorption. Interestingly, high-intensity exercise resulted in no change of the bone ultimate force or elastic modulus, and even led to increased yield stress despite the low BMD, which may be due to changes in bone structure that led to maintained or increased resistance to fracture [30].

Silva et al. found that little evidence of diminished responsiveness to 2 weeks of mechanical loading in the SAMP6 skeleton [31], and other studies revealed that 8-16 weeks running exercise improved skeletal health [32–35]. Different from previous reports, we found that only 5 weeks of medium-intensity exercise increased BMD and bone strength in the SAMP6 mice, and 9 weeks of exercise training had greater effects on the ultimate force, CSA, MAR, MS/BS and mAR. Our results suggest that the threshold of exercise duration for skeletal health is 4-5 weeks. In addition, 9 weeks of medium-intensity exercise failed to had greater effects on BMD, yield stress and elastic moduli, suggesting that exercise may maintain the balance of bone homeostasis, but can not increase bone formation unlimitedly. Moreover, this study revealed that low-intensity exercise significantly increased bone strength after 9 weeks of running exercise, with no change after 5 weeks. Previous studies indicated that exercise types with high mechanical stimulation of bone, eg. high-impact or weight-bearing exercise have highly positive effects on bone formation [36–39], thus long-term exercise is needed to obtain positive effects for low-intensity exercise. Taken together, these data suggest that exercise for senile osteoporosis prevention is mediated by a combined effect of intensity and duration, medium intensity with long- term of exercise was recommended, and low-intensity exercise may also have positive effects on bone strength and formation if it is performed over the long term.

The Wnt signaling pathway plays critical roles in osteoblasts and in Bone Mesenchymal Stem Cell (BMSC) proliferation and differentiation *in vitro* [40, 41], and downregulation of this pathway leads to reduced bone mass in SAMP6 mice [28]. Thus, we investigated the relationship between exercise and the canonical Wnt signaling pathway to determine whether the canonical Wnt signaling pathway was involved in the exercise-mediated reduction of bone loss in SAMP6 mice. Wnt1 and Lrp5 were two important protein in the Wnt/β-catenin signaling pathway [42,43]. In the present study, Wnt1 and Lrp5 mRNA expression increased after 9 weeks of medium-intensity running exercise, with no significantly change after 5 weeks of exercise, indicating that 9 weeks of exercise has stronger effects than 5 weeks on activation of the Wnt signaling pathway.

β-catenin is an another key protein in the Wnt/β-catenin signaling transduction and promotes bone formation [42, 44, 45]. Our findings showed that 5 weeks of medium intensity but no low intensity exercise increased β-catenin mRNA expression in the tibia, while 9 weeks of both medium and low intensity of exercise increased the expression. These finding contribute to the explanation of the phenomena that bone formation responses to mechanical loading is dependent on intensity and duration. Additionally, our results showed the expression of β-catenin but no Wnt1 or Lrp5 increased after 5 weeks of medium-intensity exercise, suggesting that other signaling pathways such as Akt/GSK3β might interact with Wnt/β-catenin signaling pathway at early stage of exercise-mediated bone formation[46, 47]. Collectively, These results indicate that activation of the Wnt signaling pathway is involved in exercise-induced osteoporosis prevention in this senile rodent model. In translating our findings from animal experiments to human practice, it is envisaged that the long term medium-intensity exercise should be recommended for prevention senile osteoporosis in humans, although there are some differences between mice and human.

This study has some limitations. Bone mechanical properties may have been maintained in the high-intensity group despite reduced bone density and the trabecular histomorphometric parameters due to structural changes, particularly in trabecular arrangement. MicroCT is a superior method for studying bone geometry and could improve our understanding of the response of the skeleton to exercise and mechanical loading. Therefore, future studies should use improved method to obtain a better understanding of the response of bone to exercise. Also, we detected Wnt1, LRP5 and β-catenin mRNA expression in tibia section via *in situ hybridization* staining, indicative of osteoblastic lineage. However, there are many cell types in the bone marrow that are rather difficult to be exclusively distinguished without co-staining with cell type specific markers. Besides, the protein level of Wnt signaling pathway was not investigated, which may provide a better understanding of the effects of exercise. Future study will examine the proteins expression of the Wnt signaling pathway.

In summary, our findings demonstrated that medium intensity treadmill running exercise increased BMD and bone strength by increasing bone formation in SAMP6 mice, and was more effective than low-intensity exercise. High intensity exercise can maintain or even increase bone strength, despite its negative effects on bone mass. Nine weeks of exercise was superior to 5 weeks of exercise in terms of bone strength and formation, particularly for low-intensity exercise. Exercise-induced bone formation may be mediated by activation of the Wnt/β-catenin signaling pathway. Collectively, our data indicated that the anabolic effects of exercise on skeletal health and osteoporosis prevention is dependent on exercise intensity and duration, and the Wnt signaling pathway may involved in.

## MATERIALS AND METHODS

### Animals

All animal procedures were performed according to the requirements of the ARRIVE guidelines and were approved by the Ethics Committee of Shanghai University of Sport [48]. Inbred SAMP6 and SAMR1 mice were purchased from the .A total of 48 3-month-old male SAMP6 mice were randomly assigned to two groups: a 5-week training group (*n* = 24) and a 9-week training group (*n* = 24). Each group was then divided into four sub-groups: non-exercise control group 1 and group 2 (C1 and C2, *n* = 6), low-intensity group 1 and group 2 (L1 and L2, *n* = 6), medium-intensity group1 and gourp2 (M1 and M2, *n* = 6) and high-intensity group 1 and group 2 (H1 and H2, *n* = 6). In addition, 12 SAMR1 age- and sex-matched mice were used as the homology control and randomly divided into 2 groups (R1 and R2, *n* = 6). The mice were housed under controlled experimental conditions with a maintained temperature and humidity of 22±3°C and 55-60%, respectively. All mice were provide with commercial diet containing 1.20% calcium and water *ad libitum* under a 12-h light-dark cycle (light off 19:00-7:00) and were weighed weekly on Monday morning.

### Treadmill exercise protocol

As in previous studies [49–51], the mice in all exercise groups were assigned to different speeds of treadmill running exercise representing low-, medium- and high-intensity (Table 2). In brief, all mice performed treadmill running at a speed of 8 m/min (0° slope) for 10 min/day for 6 days as a familiarization bout during the first week. In the second week, the mice in the low-, medium- and high-intensity exercise groups were assigned to run 20 min at a 0° slope at a speed of 8 m/min, 12 m/min and 18 m/min respectively. During the third week, the speed was increased by 3m/min every other day for the medium- and high-intensity groups, and the duration was increased by 10 min every other day for all groups. From the fourth to ninth weeks, the mice in the low-, medium- and high-intensity groups ran 50 min at a 5° slope at a speed of 8 m/min, 18 m/min or 28 m/min, respectively. Exercise training was performed 6 days per week with 1 day of rest throughout the program. The control mice in the C and R groups were housed under conventional conditions and did not exercise.

**Table 2:** Treadmill running protocols

	Speed (m/min)			Time (min)			Slope (°)
Intensity	low	medium	high	low	medium	High	
week 1		8			10		0
week 2	8	12	18	20	20	20	0
week 3 (day1,2)	8	12	18	20	20	20	0
week 3 (day3,4)	8	15	21	30	30	30	0
week 3 (day5,6)	8	18	24	40	40	40	0
week 4-week 9	8	18	28	50	50	50	5

### Animal tissue preparation

All mice were labeled with tetracycline hydrochloride (30 mg/kg) by intraperitoneal injection 3 and 16 days before sacrificing to fluorescently label bones. When the treadmill running program was completed (at the fifth week for C1, L1, M1, H1 and R1; at the ninth week for C2, L2, M2, H2 and R2), the mice were sacrificed after fasting overnight. All mice were anesthetized by ether inhalation, and blood samples were collected by removing the eyeball from the socket; the animals were then euthanized by cervical vertebra dislocation. The blood was suspended for 30 min and centrifuged for 30 min (2000r/min), and serum was obtained from the supernatant and stored at −20°C until biochemical analysis. The femurs and tibias of the animals were dissected with removal of the soft tissue. The right femurs were used for BMD analysis, and the left femurs for biomechanical testing. The right tibias were used to prepare undecalcified bone sections to determine bone histomorphometric parameters; and the left tibias to prepare decalcified bone frozen sections for *in situ hybridization* staining to detect Wnt1, LRP5, and β-catenin mRNA expression.

### Bone mineral density measurement

BMD was measured in the right femur by dual energy X-ray absorptiometry (Osteocore3 Digital 2D, Medilink Inc., France). The BMD values of the whole bone were expressed in absolute numbers as g of Ca-hydroxyapatite crystals percm^2^ (g/cm^2^) and were analyzed by the machine's software using an animal model (v.6.14).

### Biomechanical measurement

The three-point bending method was used to analyze the mechanical properties of the left femurs (Reger 2000, China). Both proximal and distal of femur were fixed on 2 cylindrical supports with 8-mm span; the anterior surface was placed upward with no pre-load. During the test, the load was applied at the mid-diaphysis of the bone using a probe that moved continuously down at a speed of 2 mm/min until the femur fractured. The femurs were kept moist throughout the test. All data, including load and deformation, were collected by a computer connected to the machine. The parameters measured included ultimate force (N), yield stress (MPa) and elastic modulus (GPa). The ultimate force was the force that caused the femur to fracture; yield stress was the stress at the yield point of the femur. Elastic modulus was calculated according to “the load-deformation curve” using the integrated computer software. At the same time, cross sectional area was scanned and calculated by the machine.

### Bone histomorphometry

The histomorphometric methods used in this study were described in detail by Li [15]. In brief, the right tibias were dehydrated in increasing ethanol concentrations; xylene was used as a transparent agent, and the tibias were embedded in glycol methacrylate. Undecalcified bone sections were cut at thicknesses of 5 μm and 10 μm. After deresination and staining with toluidine blue, the 5-μm sections were viewed by microscopy. The 10-μm sections were directly examined by fluorescence microscopy. Leica QWin image analysis software was used to obtain the following histomorphometric parameters: BV/TV, ES/BS, MS/BS, MAR, O.Th, mAR) and BFR.

### Tibia Wnt1, LRP5, and β-catenin mRNA expression

The left tibias were fixed in 4% paraformaldehyde for 24 h, fully rinsed with 0.1 M PBS (pH 7.2 to 7.4), and decalcified for 3 weeks in 10% EDTA/0.1 M PBS (pH 7.2 to 7.4) at 4°C. The buffer was replaced with fresh buffer every other day. After dehydration in 15% sucrose solution/PBS, the decalcified bones were embedded in OCT, and 10-μm sections were prepared (Leica RM2235, Germany). Sections were incubated in SSC twice in 70°C for 30 min, rinsed with distilled water and treated with pronase (0.125 mg/ml in 50 mM Tris-HCl, 5 mM EDTA, pH 7.5) for 10 min. After digestion, they were rinsed with distilled water, fixed in 10% formaldehyde, blocked in 0.2% glycine, rinsed with distilled water, rapidly dehydrated in a series of graded ethanol solutions, and air dried for several hours. Finally, the sections were hybridized with digoxigenin-labeled antisense probes or sense probes as negative controls. The DAB method was used after the sections were washed twice with PBS for 5 min. The mRNA sequences corresponding to the primers for the anti-Wnt1, LRP5, and β-catenin probes were as follows:

Wnt1: (1)5′-CGAGA GTGCA AGTGG CAATT CCGAA ACCGC CGCTG-3′;

(2)5′-CGGGA CCTAC GCTTC CTCAT GAACC TTCAC AATAA-3′;

(3)5′-TGCAC CGTGC GCACG TGTTG GATGC GGCTG CCCAC-3′;

LRP5: (1)5′-ATACC ACTAT ATCCA TGTGC TGGAC CAGAA-3′;

(2)5′-GAAGT GGAGG TCGTG GAGAT CATTC AGGCC-3′;

(3)5′-CAGGA TGTGT ATGTG CTGTC GGAGC AGCAG-3′;

β-catenin: (l)5′-ACTCA AGCTG ATTTG ATGGA GTTGG ACATG-3′;

(2)5′-GGGTT CAGAT GATAT AAATG TGGTC ACCTG-3′;

(3)5′-TGCCT CCAGG TGACA GCAAT CAGCT GGCCT-3′;

Leica QWin image analysis software was used for analysis. A positive reaction was indicated by brown coloration of the cytoplasm. More than five high-power fields (× 400) below the epiphyseal plate within the medullary cavity were randomly selected and blindly analyzed.

### Serum analysis

Serum ALP, OCN and PTH were measured by enzyme-linked immunosorbent assay (ALP and OCN using Xitang ELISA KIit, China; PTH using ELISA Kit, IBL International GmbH, Hamburg, Germany).

### Statistical analysis

All data are presented as the mean ± standard deviation, and all figures were prepared using GraphPad Prism 5 (version5.01, GraphPad Software, Inc.). Two-way ANOVA was performed, followed by the Bonferroni *post hoc* test for comparisons of the different exercise intensities and of 5- and 9-week exercise training. For statistical evaluation, significant differences were considered at *p < 0.05*. According to power estimation (G*power Version 3.0.10, Germany), five mice for each group would have been required for 90% power as the sample size calculated. All statistical analyses were performed using the statistical software SPSS 13.0 for Windows (version 13; SPSS Inc., Chicago, IL).
